# Supplementary material for: Assessing the Association between Important Dietary Habits and Osteoporosis: A Genetic Correlation and Two-Sample Mendelian Randomization Study
Source: Nutrients. 2022 Jun 27;14(13):2656. doi: 10.3390/nu14132656 (PMC9268345; doi:10.3390/nu14132656)

Assessing the association between important dietary habits and osteoporosis: A genetic correlation and two-sample Mendelian randomization study

Supplemental materials

Supplemental Figure S1:

Forest plots of variant specific inverse variance estimates for the causal association between OP and candidate dietary habits.

Supplemental Figure S2:

Scatterplots of the causal relationships between OP and candidate dietary habits.

Supplemental Figure S3:

Funnel plots of the causal association between OP and candidate dietary habits.

Supplemental Figure S1:

Forest plots of variant specific inverse variance estimates for the causal association between OP and candidate dietary habits.

A:OP and candidate PC17

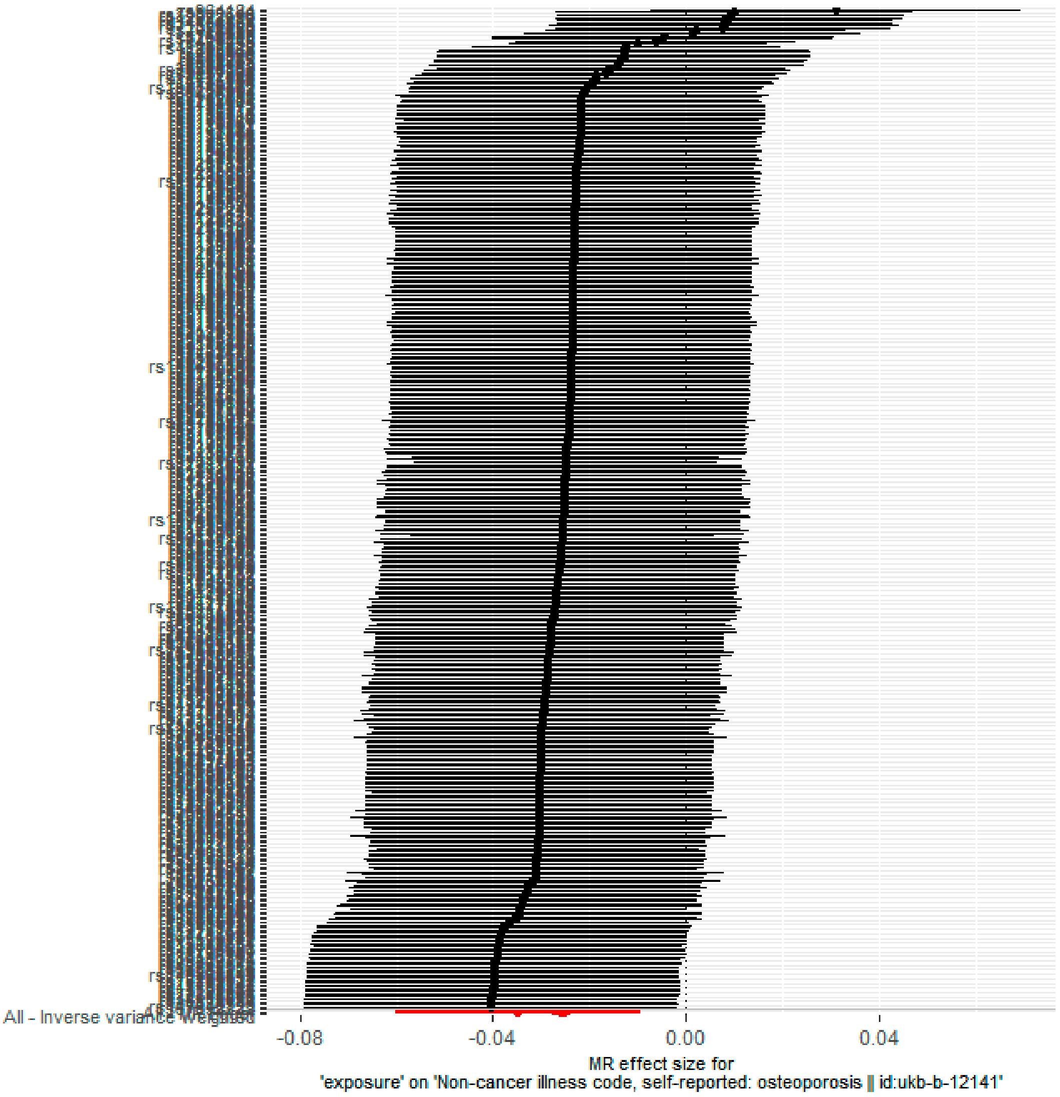

B:OP and candidate PC35

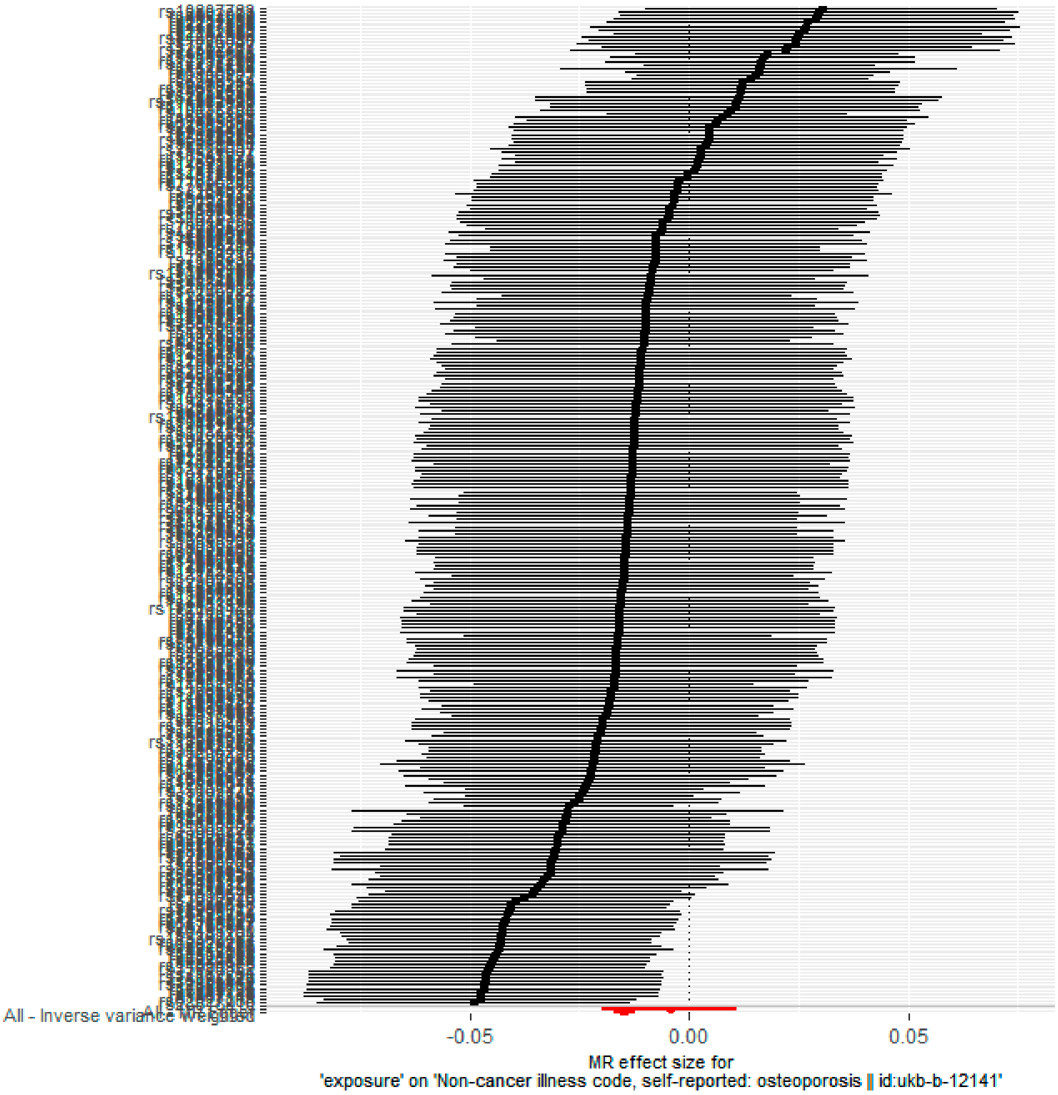

C:OP and candidate PC36

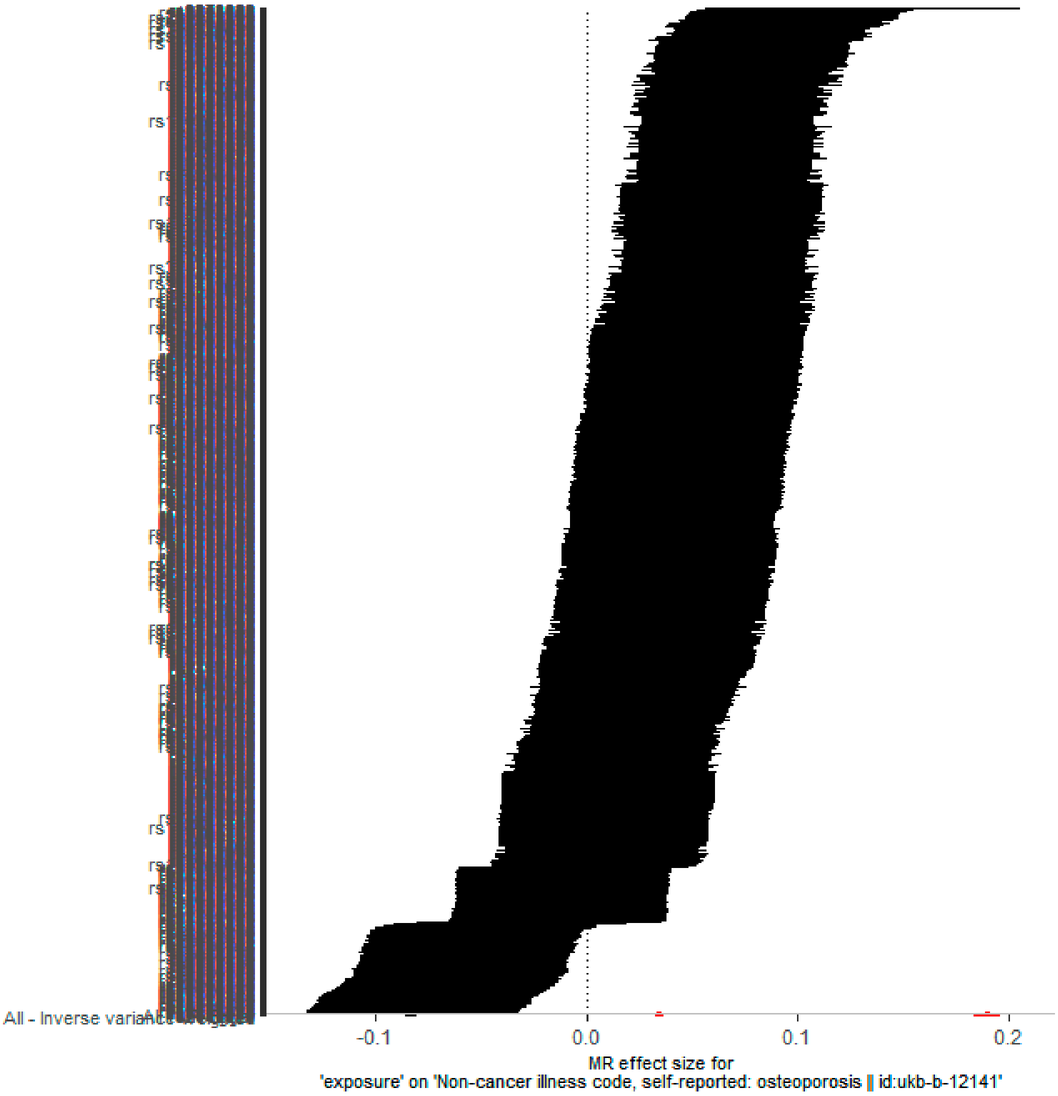

D:OP and candidate PC39

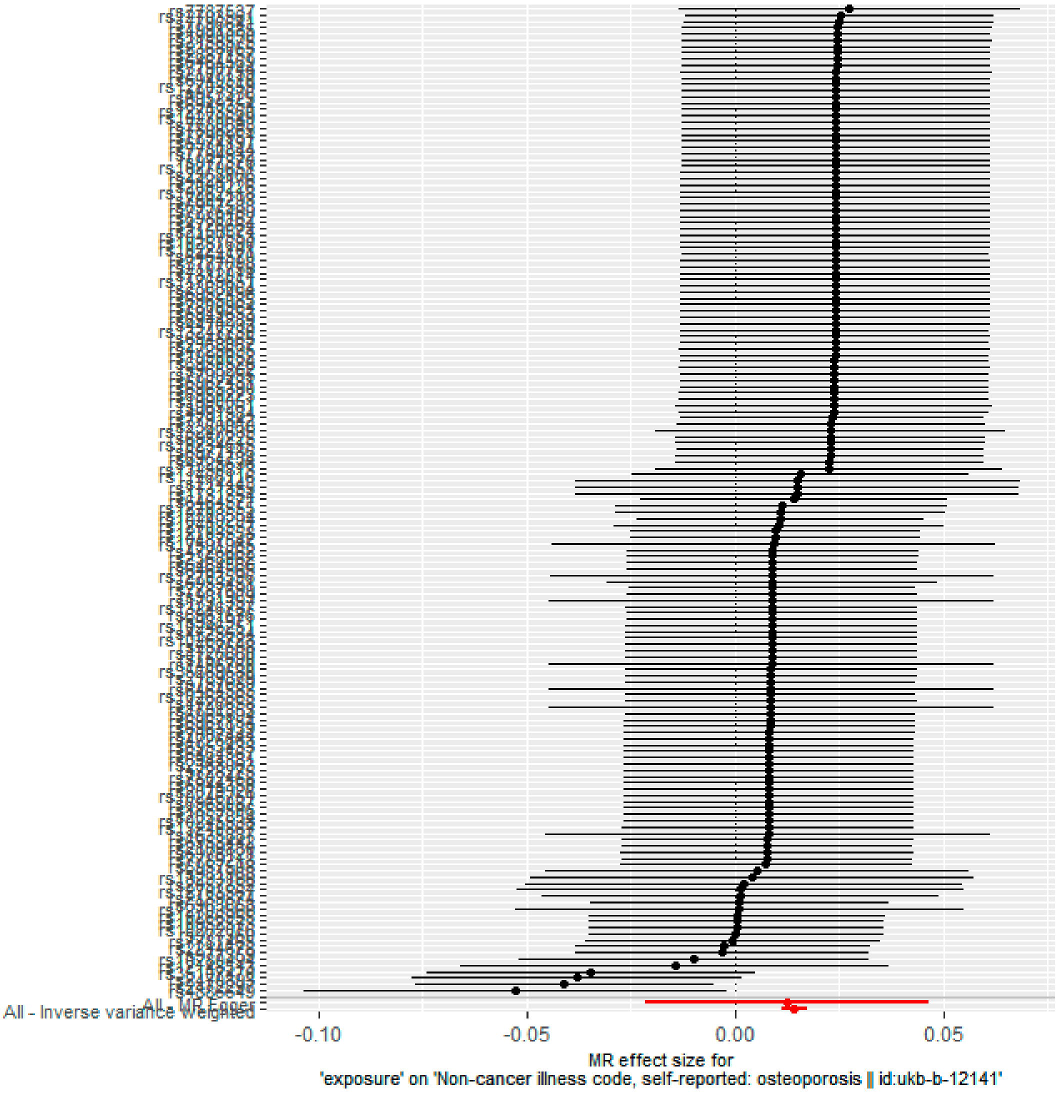

E:OP and candidate tablespoons of raw vegetables per day

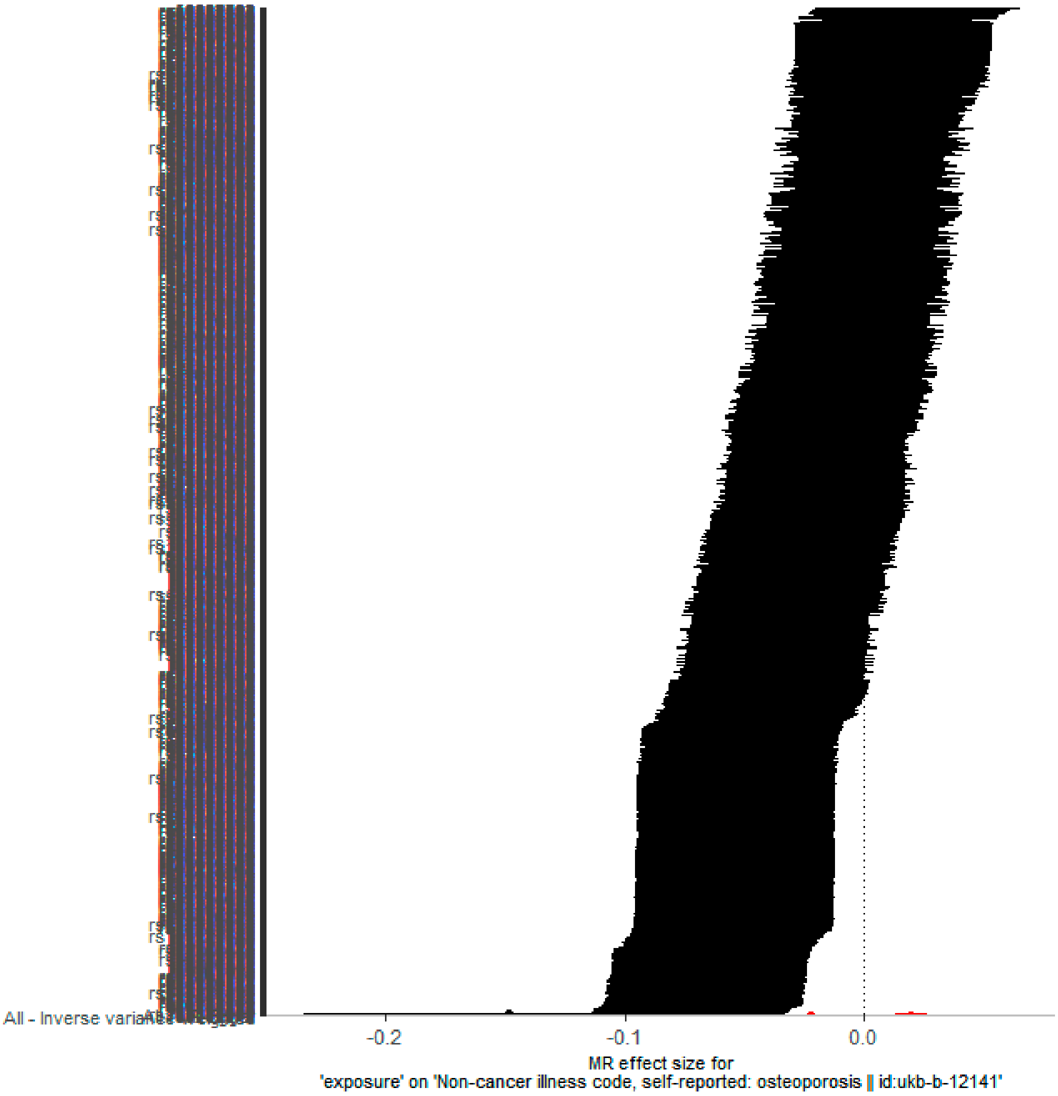

Supplemental Figure S2:

Scatterplots of the causal relationships between OP and candidate dietary habits.

A:OP and candidate PC17

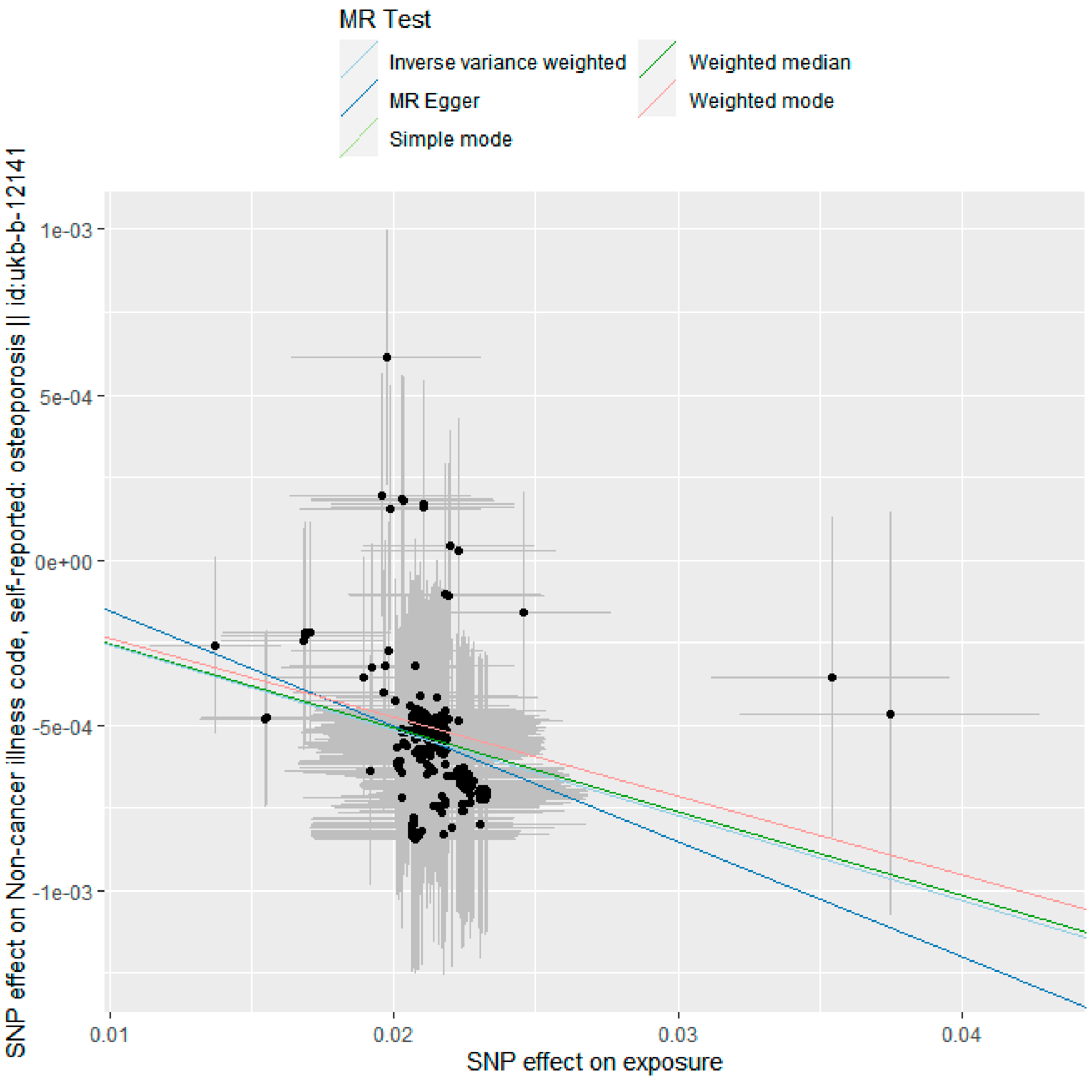

B:OP and candidate PC35

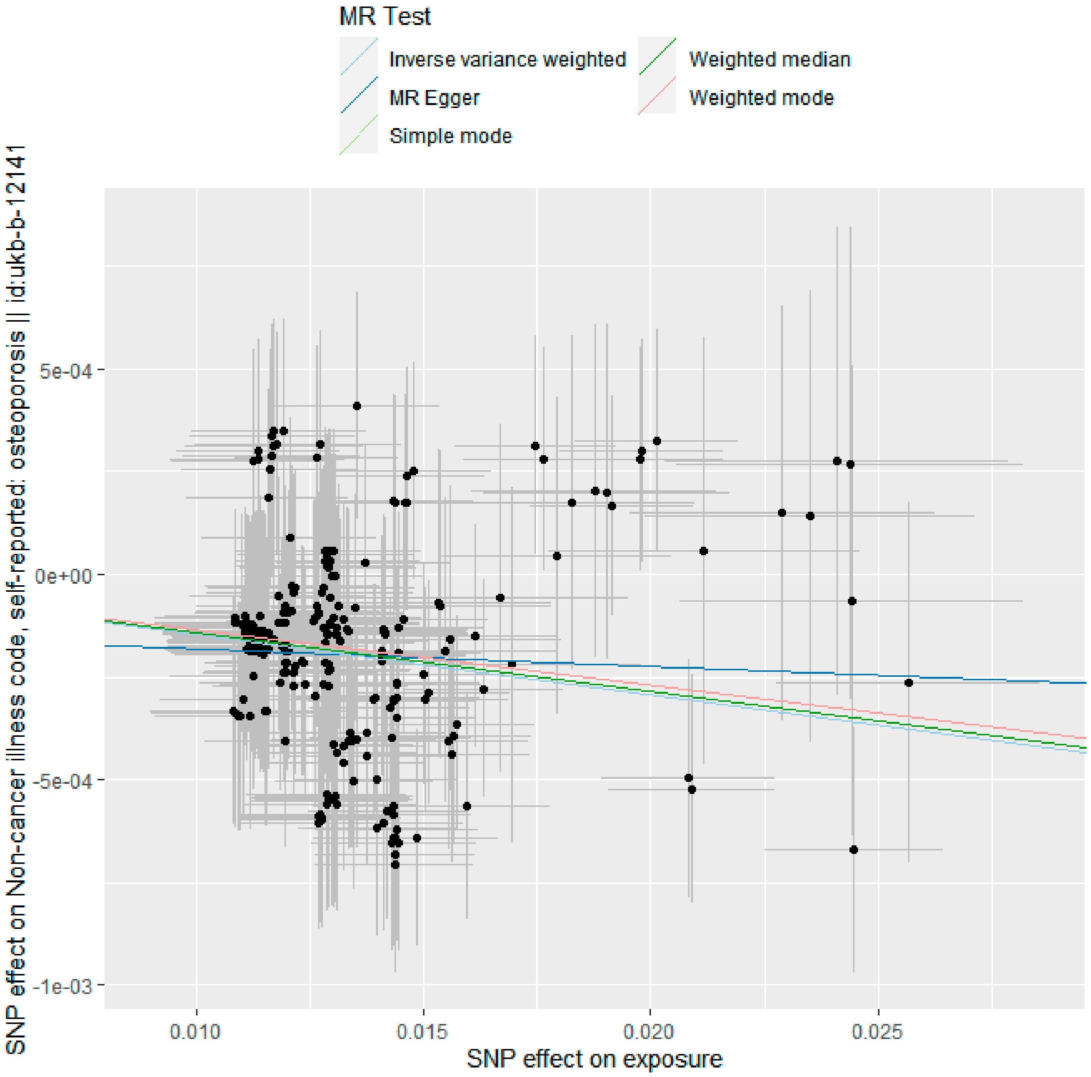

C:OP and candidate PC36

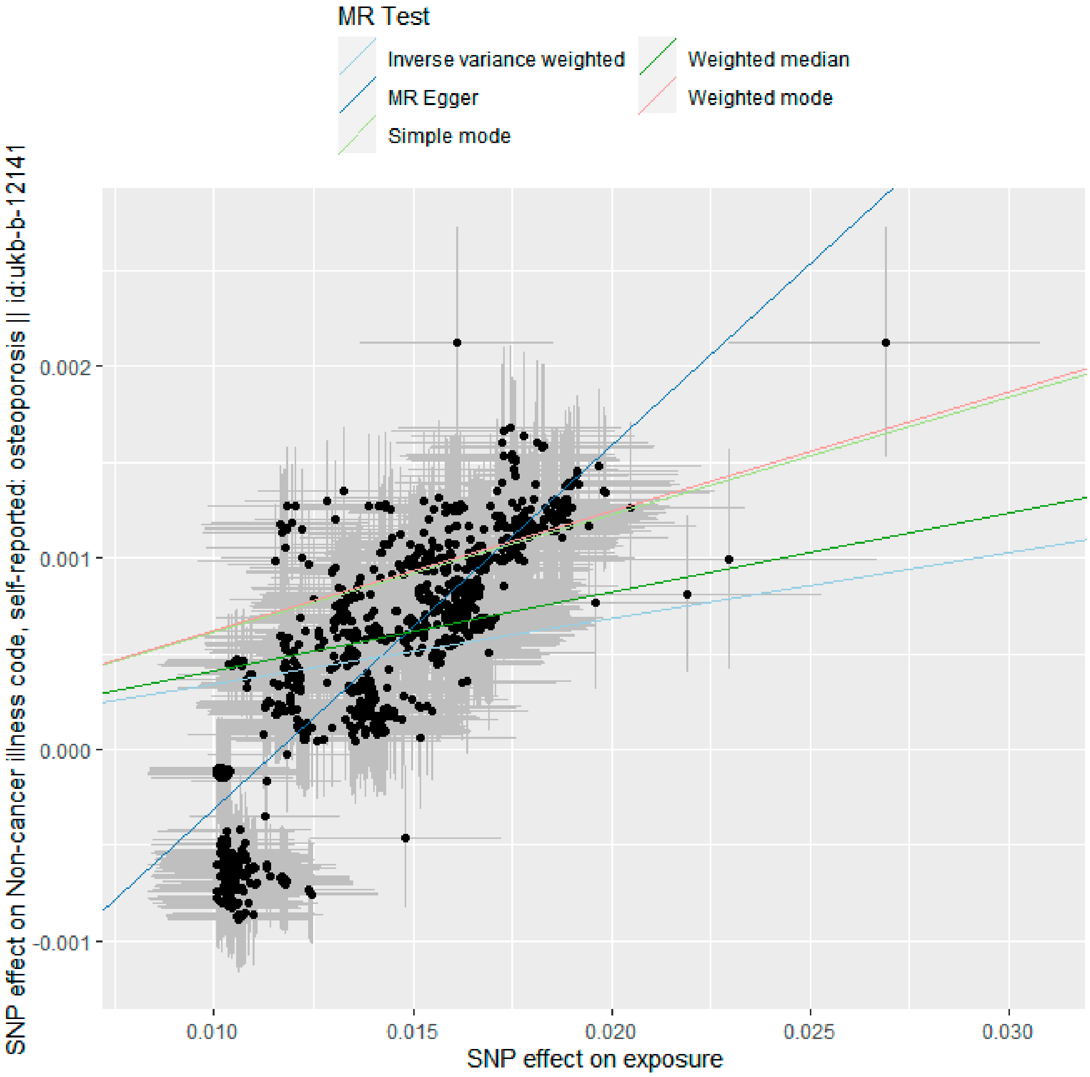

D:OP and candidate PC39

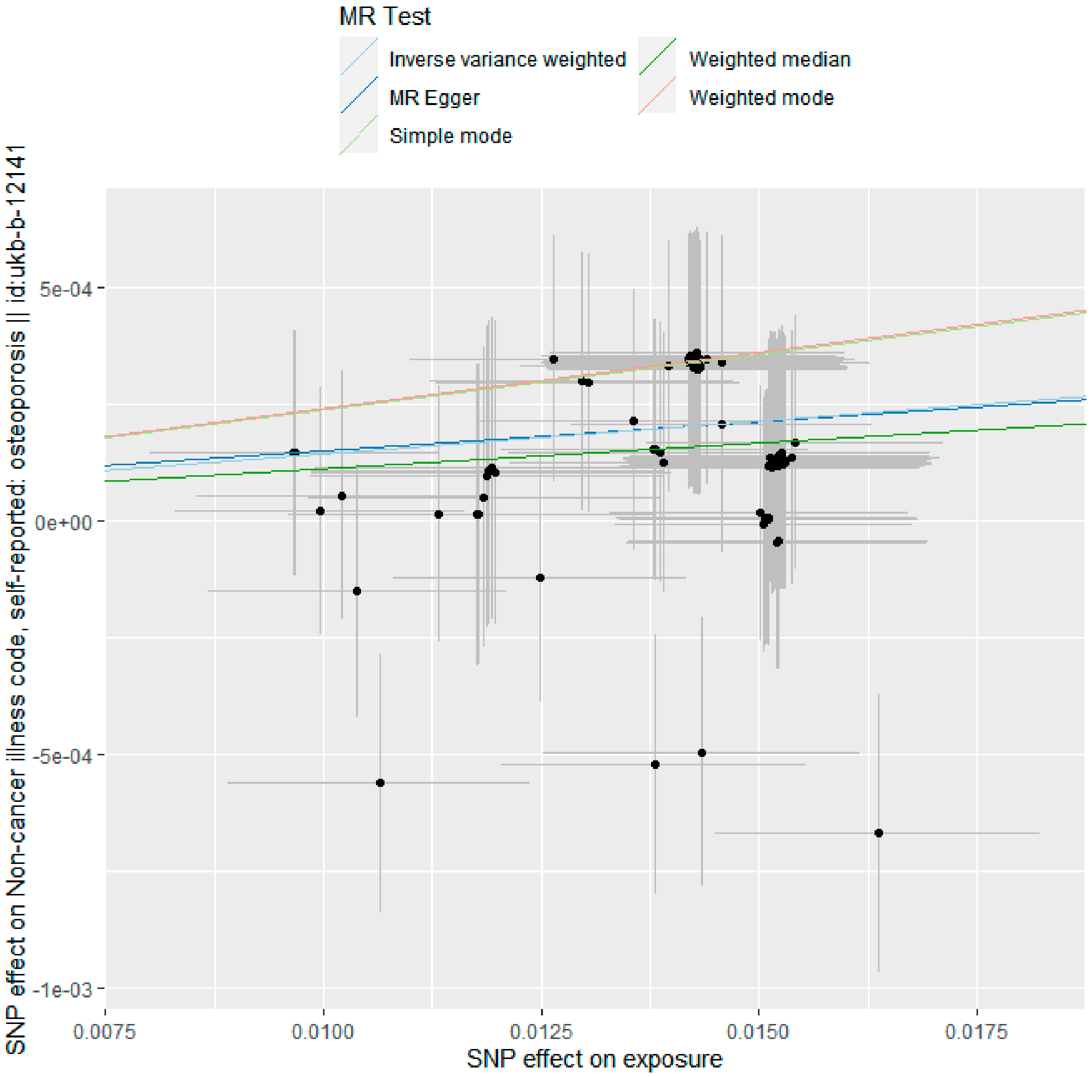

E:OP and candidate tablespoons of raw vegetables per day

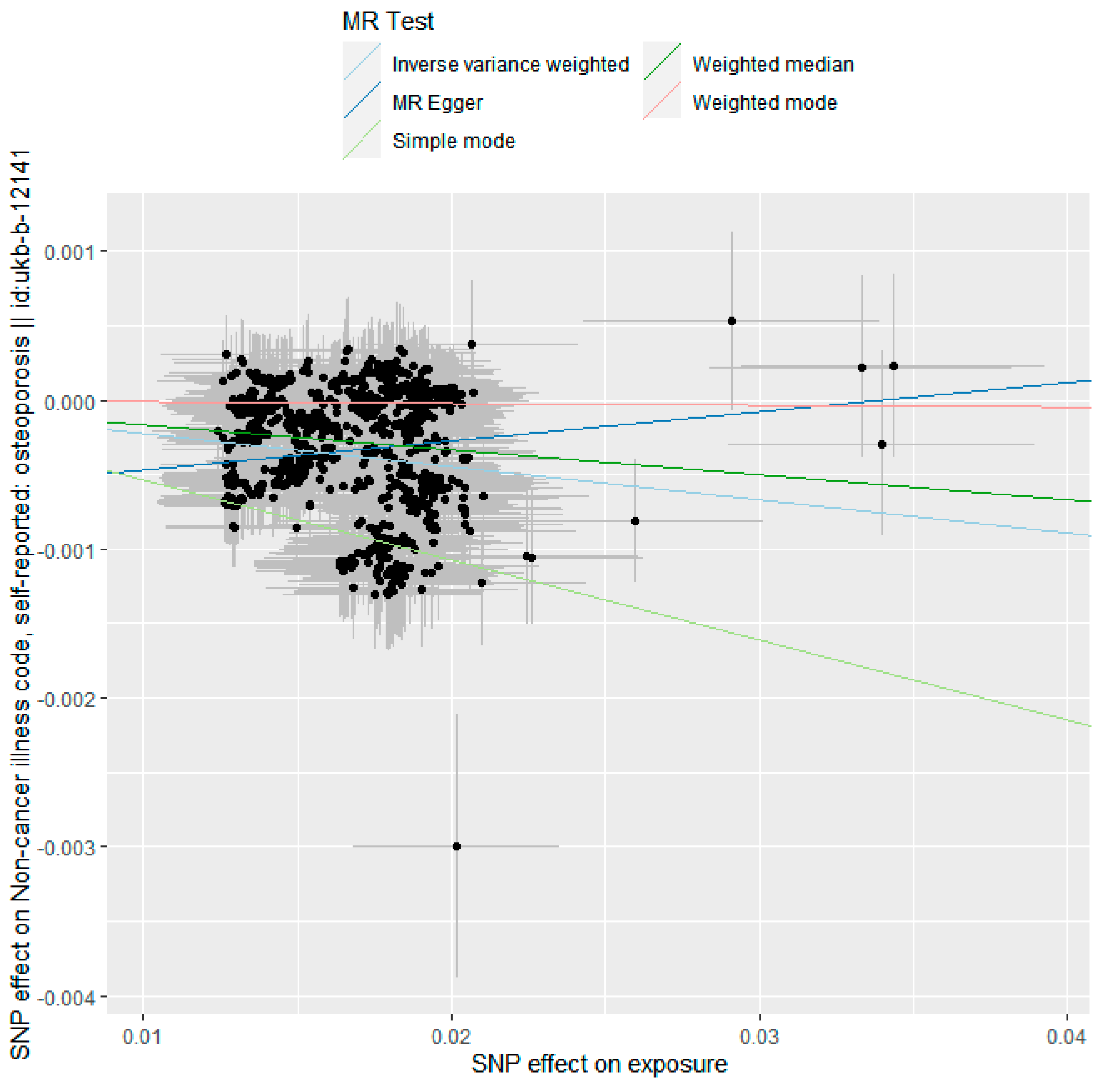

Supplemental Figure S3:

Funnel plots of the causal association between OP and candidate dietary habits.

A:OP and candidate PC17

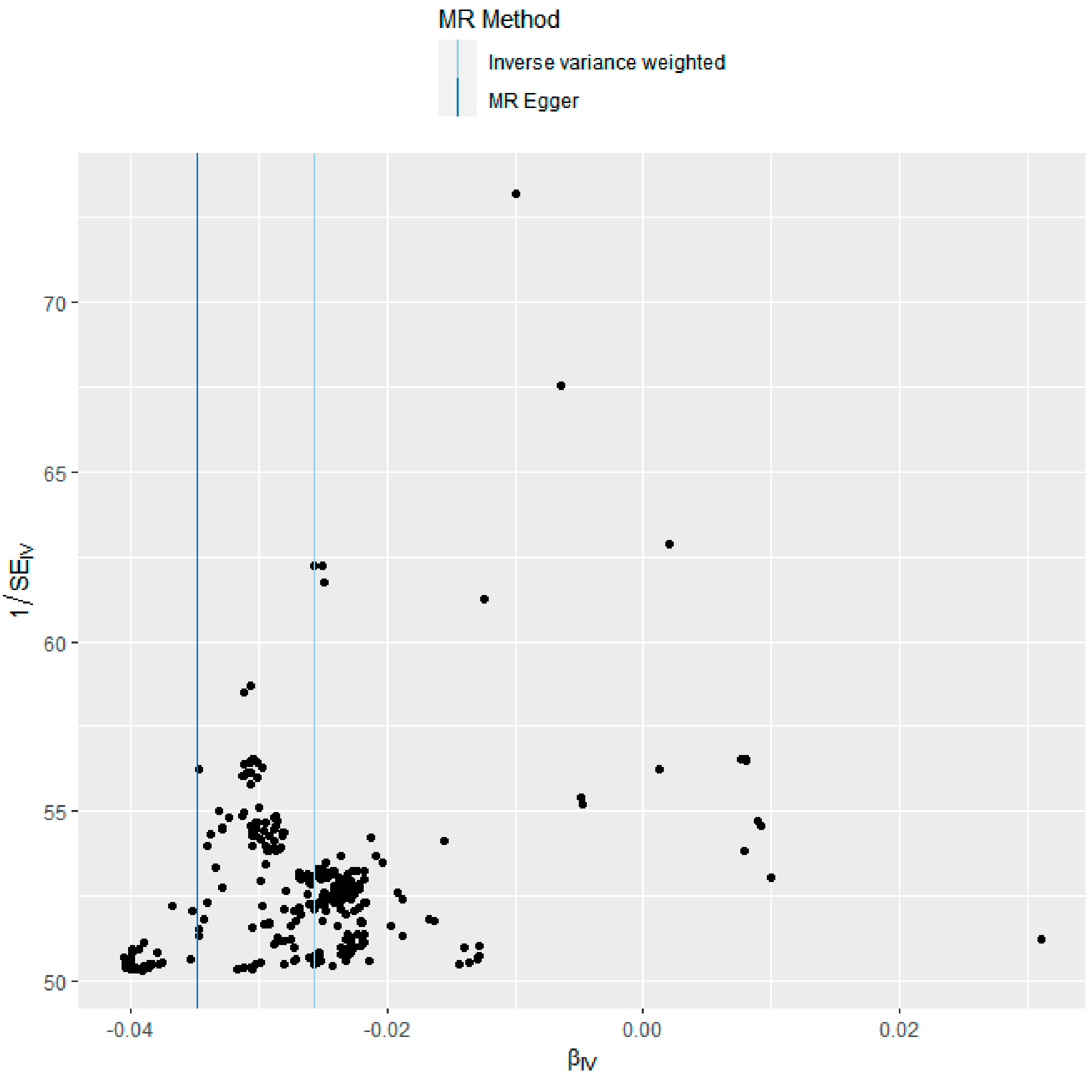

B:OP and candidate PC35

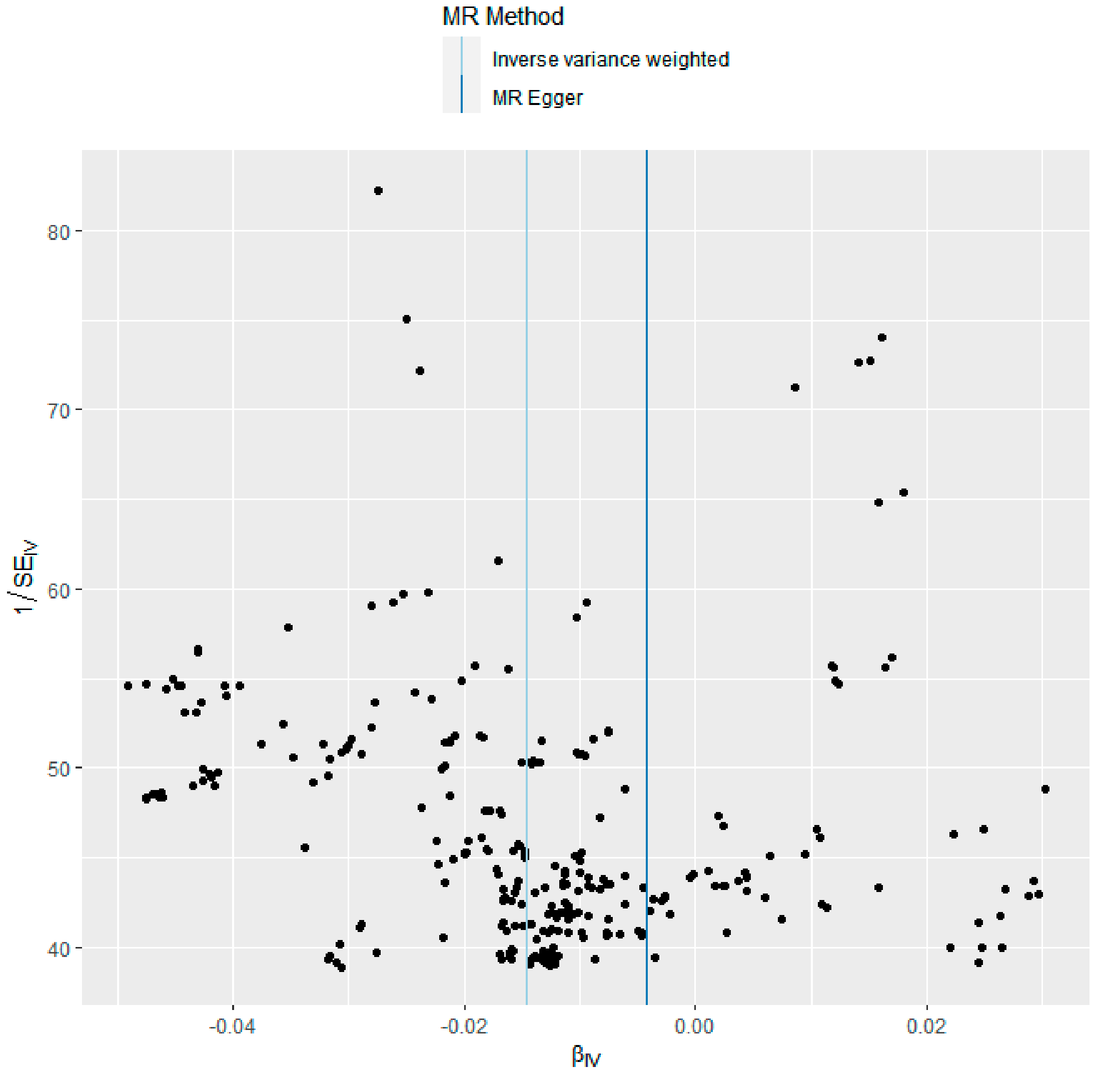

C:OP and candidate PC36

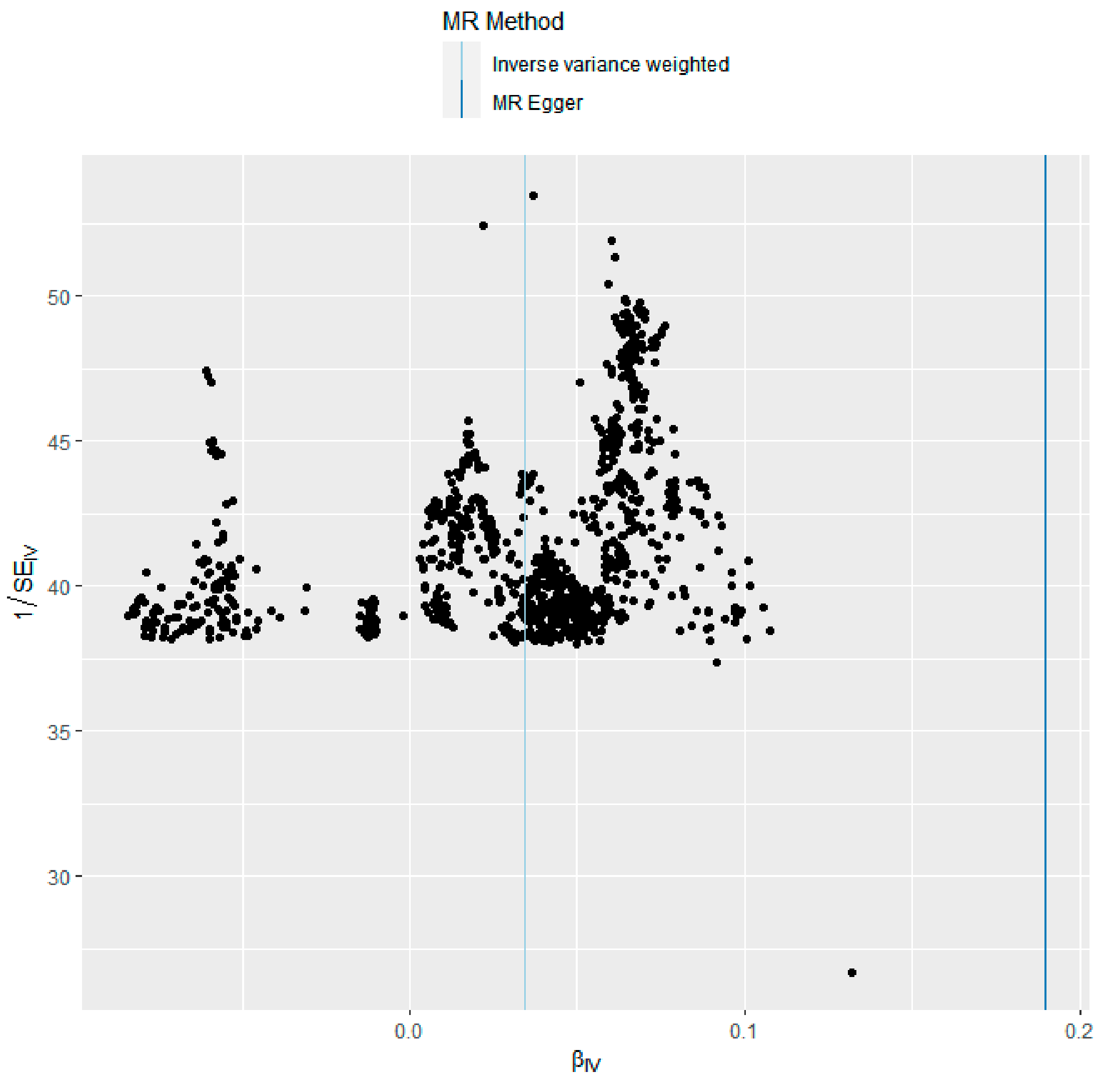

D:OP and candidate PC39

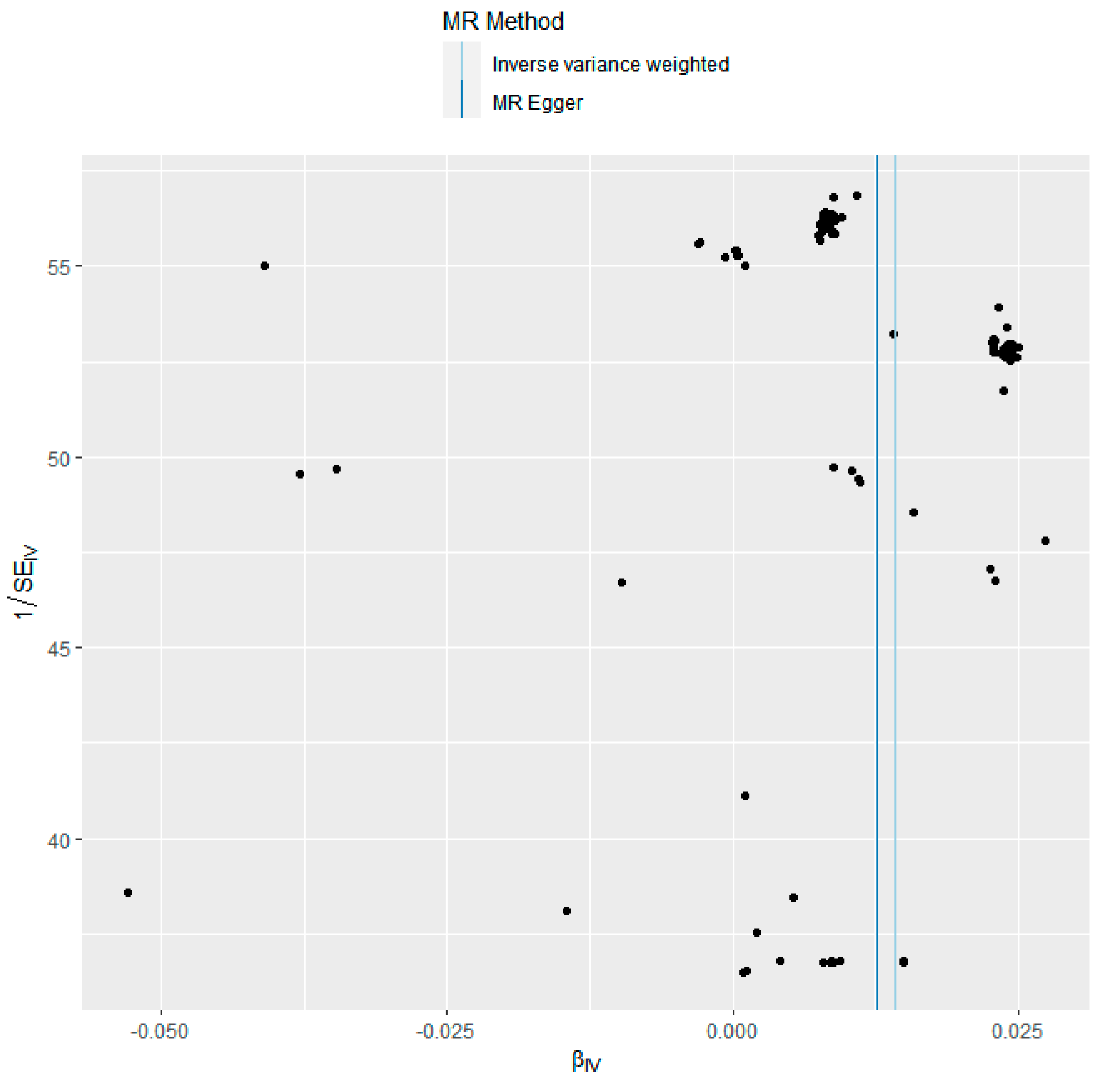

E:OP and candidate tablespoons of raw vegetables per day

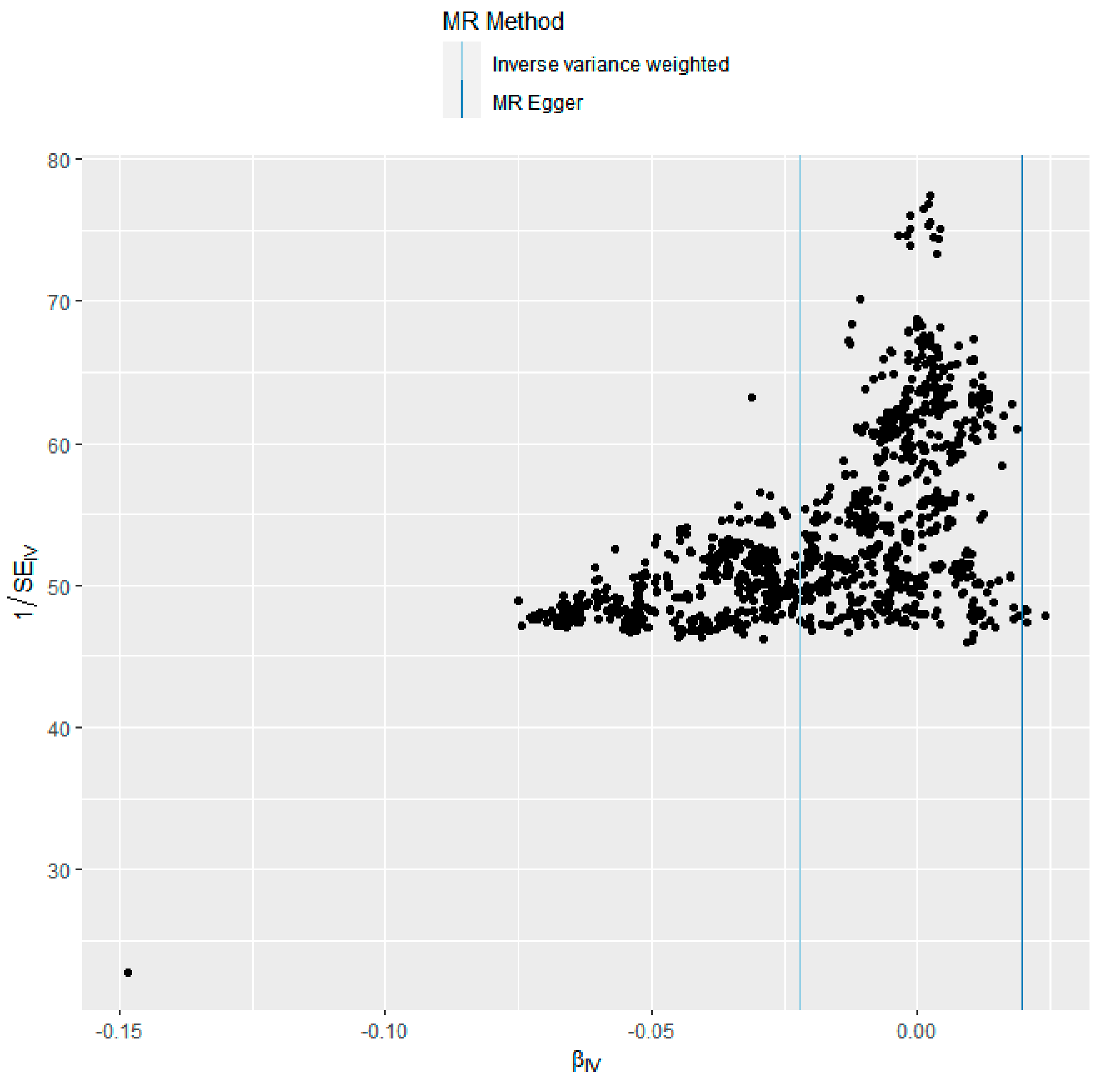

Supplement: Supplementary file 1 [file nutrients-14-02656-s001.zip › nutrients-1758006-supplementary.pdf]
